# A Memoir on Obliteration of the Bronchiæ

**Published:** 1841-05

**Authors:** A. C. Reynaud

**Affiliations:** Formerly resident in the Hospital; late Chief of Clinical Medicine of the Paris Faculty; Physician at Puy, &c.


### WESTERN JOURNAL

OF

### MEDICINE AND SURGERY.

MAY, 1841.

### Art. I.—*A Memoir on Obliteration of the Bronchiæ: By* A. C. Reynaud, M. D., formerly resident in the Hospital; late Chief of Clinical Medicine of the Paris Faculty; Physician at Puy, &c. Translated for the Western Journal of Medicine and Surgery: By John A. Warder, M. D., of Cincinnati.

(concluded.)

### *State of the neighboring Bloodvessels.*

The bloodvessels were not found closed, except where an extreme dilatation of the bronchiæ had preceded their obliteration: by insufflation, indeed, we were able to follow them as great a distance as in a sound lung, but this was not the case with the vessels that accompany the terminal fibrinous branches of the obliterated bronchiæ; air could not be forced into them, and the absence of circulation was indicated by their black color and hair-like appearance. It may be here mentioned, that the filaments of the obliterated bronchiæ, as well as their terminal enlargements, were generally of a dark, black color,—which may be owing to the discoloration of the solid particles of blood after the circulation has ceased, and is the same observed in so many chronic alterations of the lungs, especially at their summit.

Our researches have not been directed to the nerves and absorbents of the lungs, in these cases; for their exceeding delicacy prevents our following their branches distinctly, into the altered parts of the organ.

*State of the Cellular Tissue.*

A remarkable appearance was observed where the last filaments of an obliterated bronchia terminated: as elsewhere, when parts become atrophied or diminished in volume, this tissue tends to occupy their place, so here it keeps apart the bronchial ramifications, which, when distended with air, constituted the whole pulmonary fabric. Thus the various elements of the primitive structure of the organ, may be very distinctly observed, not merely from their peculiar form and isolation, but also by their black color, which contrasts strongly with the greyish hue of the cellular tissue. This substance was also much softer than other parts, and drawing slightly upon one of the trunks of the terminal filaments, we perceived a number of furrows, caused by the pressure on an unresisting substance, like those seen in dissecting the final ramifications of the nerves, when the eye has lost sight of them, they are again brought into view by exerting a slight traction on their trunks. As it was more readily torn than the fibrous threads, the cellular tissue could be easily removed by scraping with the handle of the scalpel, while the ultimate bronchial and vascular filaments were so exposed that they could be seen floating under water, or in the current of air from a small blow-pipe.

*Causes of Obliteration.*

With our views of the physiological properties of the mucous membranes, and their slight tendency to form adhesions, we are surprised to find the bronchiæ so often obliterated: indeed, we may suppose that their lining membrane, and especially that part of it which performs the physiological act of respiration, differs from the other mucous membranes, as it is much thinner and more pellucid than the layer which lines the commencement of the air passages, and the digestive tubes: mere continuity does not appear to me sufficient proof of an identity of character.

In the smaller bronchiæ we find neither villosities nor follicles, and their natural secretion is serous, not mucous. We know that certain pathological conditions, such as continued inflammation, will develope the structure of mucous membranes, and that products, analagous to those of mucous surfaces, are frequently observed: but, the pathological condition has great power of transformation—a serous cyst, whose natural function was to secrete an aqueous fluid, may be converted into a mucous cavity capable of secreting mucous, or pus; and in the same manner all the organs, with any given organization, may give rise to tissues or organic products of different characters.

Besides, the lining membranes of the air passages is much more frequently affected with adhesive inflammation than the ordinary mucous tissues; and I have reason to believe that the bronchial extremities are frequently in an analagous condition. I have found many cases of acute pneumonia with hepatization, where the inflammation had resulted in false membranes in all the lesser bronchiæ, filling their cavities more or less completely, and leaving the surrounding parts untouched.

If to these data, based upon the pathological characters which belong especially to the serous membranes and the internal coats of vessels, we add the fact of the frequent obliterations of bronchiæ, of vessels of serous cavities, we shall have some reason to suppose that among these several parts, there is as complete an identity as their various functions will admit. If this analogy be well founded, we may conclude, *a priori*, that inflammation of the bronchiæ, especially the small ones, may cause their obliteration in the same way that a blood vessel is occluded by a corresponding attack of inflammation:—the following fact will place this obliterating influence of inflammation beyond a doubt:

A man aged about twenty-five, was attacked with small-pox; the eruption had run its ordinary course, the scabs were detached, leaving scars on the face, when a pneumo-thorax of the right lung ensued. Its presence was indicated during life, by extreme dyspnœa, a remarkable dilatation of the affected side, and a greater ressonance than natural. The patient was much emaciated and had a cough, but M. C. A. Louis did not suspect the presence of phthisis except from the perforation of the pleura. The patient soon died suddenly; a considerable amount of air was found in the right pulmonary cavity, the lung was pressed closely against the vertebral column and retained there by soft, recent false membranes: a perforation of the pleura, leading to a collection of pus, existed at the base of the organ. There were many enlargements on the surface, and a number of little tumous were perceptible on the touch, conveying the sensation transmitted by a lung impacted with tuburcles: indeed tumours were found within the organ, but though apparently tuburcu-

.ar, they had an unusual aspect. A careful dissection of the small bronchiæ showed them to be very red, further along their surface was covered with a slight layer of lymph, and beyond this, their cavity was filled with the same matter, up to their final terminations which appeared granulated. The accompanying blood vessels were black as charcoal, while the pulmonary parenchyma appeared, up to the limits of the alteration, of a marked reddish brown. Neither induration nor the semi-transparent grey infiltration were observed. At the same time, little cavities filled with pus, existed throughout the organ, as numerous at the base as at the superior portion. At the summit, one of these cavities was larger than the rest, and filled with what appeared laudable pus—it was lined with a yellowish white membrane half a line thick, separating it from the lung which was of a reddish brown for half a line, beyond which it was of the natural rosy hue and entirely exempt from tuburcles. A bronchia of considerable size, larger than natural, thickened and of a bright red, ran towards this cavity, but did not enter it; indeed the membrane above mentioned, extended into the tube for six or seven lines, forming a firm layer that was readily detached from the bronchial membrane. This plastic matter had the same characters as that contained in the little bronchiæ, which, by their union, formed the tuburculous looking masses which we had observed. I shall not here insist upon the difference between this lung and those affected with tuburcle; suffice it to say, it was striking, though, without a careful examination, the distinctive characters could not be precisely defined.

The purulent sac appeared to be nothing more than an abscess: the matter in it was true pus, perfectly thickened, destitute of tuburculous clots or detritus. The soft, white, unorganized substance which lined the cavity, was precisely like that found in an abscess in the parenchyma of an organ: neither induration, tuburcular granulations, nor the semi-transparent grey infiltration were found around it—a red inflammatory circle was all that separated the sound from the diseased portions. Finally, the prolongation of this false membrane into the bronchiæ, manifestly inflamed, but not ulcerated, conclusively demonstrated both its characters and nature.

Had the patient survived, what would have become of this abscess? Would the pus have found exit; would the oblit erated bronchiæ have been rendered permeable by the absorption of the plastic matter; or would the obstruction have remained permanent, and the abscess have become encysted? I shall not here discuss these questions, as the case was merely introduced to prove that obliteration may occur from false membranes, produced by evident inflammatory action.

It will be readily conceived that the bronchiæ should be obliterated when the circulation of air is obstructed, as is observed in an artery from which the blood has been excluded. But in the arrangement of the bronchiæ there is no anastomosing between the branches, so that where an obstacle exists, all the further portion of the tube must become impermeable: this cessation of the influx and efflux of air, which constitutes the circulation in the bronchiæ, may be followed by the entire absorption of the air contained at the moment of obliteration, when the shrinking of the walls upon themselves with consequent adhesion, aided by pressure of the surrounded parts, will ensue. That the air contained in a lung may be thus absorbed, after its escape is prevented, will be incontestibly proved by a fact to be mentioned in this memoir; though we do not consider it demonstrated that the obliteration of several branches will result from an obstruction at first limited to a point in the trunk whence they arise. Hoping to throw some light upon this difficult point we shall cite the following case, which is, besides, a remarkable form of the lesion. In the body of a female who died under the care of M. Louis at La Pitiè, I found one of the lungs presented these characters: The principal bronchiæ of the superior lobe, five lines in diameter, was obliterated a little more than two inches from its origin; instead of being a solid cord beyond the obliteration, it was dilated into an oval sac and filled with a semi-fluid, yellowish white substance somewhat like tuberculous matter mixed with mucous. At some distance, the subdivisions were obliterated a few lines from their origin, and degenerated into fibrous cords that were again divided into solid threads terminating near the pleura.

Below the obliteration of this bronchia, we could nowhere see any of the little orifices leading into the secondary branches, but in their stead were imperforate circular depressions. From the exterior surface, at all the corresponding points, solid fibrous cords could be traced far into the substance of the lung. Two of these cicatrices only could be perforated by a pin; the higher of these opened into a fusiform portion of a bronchia about twelve lines long, and four in diameter at its largest part. From its extremity started three little ramifications, which were soon obliterated. This cavity contained no foreign matter and was permeable to air.

The second opening was much lower and led into a fusiform cavity of considerable diameter. From its proximal extremity several branches were given off, with natural calibre, and permeable to the air, even to their vesicles; others were funnel-shaped, forming a cul-de-sac, and continued as solid fibrous cords: one of them, obliterated four lines from its origin, instead of being continued as a solid cord, had its cavity reproduced and considerably dilated for eight lines in extent, and again obliterated in all its branches; this last cavity was filled with matter similar to that above described.

Let us observe the connexion between these changes and how some may induce others: it is very probable that the starting point existed in the first bronchia, and that the primitive alteration was a contraction, whence resulted the obliteration of a great many orifices leading into the bronchiæ. We cannot indeed admit that the orifices were closed in consequence of the obliteration of these bronchiæ, since we find the same occlusion at the origin of a branch which is very much dilated and filled with a peculiar matter. On the other hand we find two of these orifices in progress of obliteration, scarcely admitting the point of a pin, but each opening into a dilated bronchial cavity and permeable to air. We cannot admit that the obliteration of these tubes was the cause of the occlusion of their openings into the principal bronchia. But if we admit that this contraction of the orifices was primary, it will be easily conceived how, before it ended in complete occlusion, it might have induced the dilatation of the tubes—by opposing a barrier to the escape of the air at each expiratory effort. When the occlusion was effected, the dilated cavity must needs appear to be a sac, in which the products of secretion must collect, and the final ramifications would be obliterated when the contained air was absorbed. According to this view of the facts, the final degree of obliteration should be the closure of these dilated trunks—after the gradual absorption of the secreted matters. Indeed we have reason to believe this is effected, and that it is by the same process that in some rare cases, tuberculous cavities seem to become cured, or cicatrized. These interpretations are given as conjectural, they have not warped our judgment in observing facts: in this as in all other cases, precision has been our chief ambition.

When speaking of the lesions of the lung that frequently accompany this affection, I said that we often observed it contemporaneously with tubercular disease. We frequently find, in different parts of the lungs of phthisical subjects, but especially near the summits, encysted or free masses, of a tubercular aspect, resembling calcareous matter diluted with water, or like dry chalk: these are of various size and consistence, some as large as a pea, others the size of a nut, and often, when at the summit, accompanied by a remarkable crimpling of the external surface. In almost all cases of this kind which we have carefully examined, the bronchiæ running to these altered portions of the organ were obliterated to some extent.

We shall cite a remarkable example: At the summit of one of the lungs of a female who died at La Pitié, from a cerebral attack, there existed a marked crimpling of the pleura, and at the corresponding part of the lung, was a cavity with firm greyish walls a quarter of a line thick, as large as a nut, and filled with matter resembling moistened plaster. The side of this cavity next the root of the lung was funnel-shaped, the cul-de-sac terminating in a fibrinous band the size of a crow quill, and this was the continuation of a considerable bronchial branch running in that direction and obliterated rather more than half an inch from the cavity: many neighboring bronchiæ were obliterated. Near the root of the lung there was a little fibrinous cord which arose from a large bronchia, and soon terminated in a cavity the size of a kidney-bean, which contained matter similar to that at the summit of the lung.

In a second case, there was a cavity at the centre of the inferior lobe of one of the lungs, the size of a very small filbert, with fibrinous walls, filled with old tubercular matter. A bronchia ran towards, but was obliterated within two lines of it: within a short distance of its insertion and running in the same direction, was another bronchia that was dilated, and filled, nearly to the surface of the lung, with matter, similar to that in the cavity. The walls of this tube were thickened, the internal surface white and fibrinous. The branches were oblitered some lines from the periphery of the lung and formed solid cords. This individual had been phthisical, and we found tubercles, both crude and softened. Whatever we may suppose to have been the nature of the primary alteration that caused this cavity and collection of matter, it appears evident, that having no exit in the natural direction, the tube being obliterated, the matter flowed into another branch, perhaps the continuation of the first, and caused its distension to so remarkable a degree.

It might perhaps have sufficed to have described this species of obliteration, and its connection with the alteration of the lung just mentioned, but we should investigate the cause of this lesion, and this discussion requires us to make some observations on the nature of these accidental matters contained within the pulmonary tissue. It is ever difficult to ascertain the origin and nature of any organic lesion, when we can only see the final results, and when the primary characters have been long effaced. Anatomy, when deprived of the aid of symptomatology, must depend upon its own discoveries. To recognize the more or less frequent coincidence of one lesion with another, to define their points of contact, to seek out carefully all their degrees, in order to observe the different points of relation, and connexion, are the only means of induction which we are allowed to employ. These chalky masses and collections of calcareous matter, resembling partially dried tubercles, are frequently found in phthisical lungs, especially in advanced age. Still, they are frequently found without this disease of the bronchiæ, and, during a year spent in the Hospice de la Vieillesse, we were struck with the frequency of this alteration of the lungs. My old colleague and friend Dr. Secrétain, who was simultaneously engaged in another department, has made the same remark in his inaugural thesis.

This alteration was generally found in the summit of the lungs, while they, at the same time presented externally, considerable puckering; the neighboring pulmonary tissue was somewhat black, indurated, and connected by adhesions to the thoracic cavity. This accidental matter was always contained in a kind of sac, the walls of which were thickened, somewhat smooth internally and apparently fibrous, and colored to some extent by the black pulmonary substance.

The contents presented various appearances, sometimes resembling tubercular matter, only drier and more friable: at others, looking like the detritus of tubercular matter mixed with water, not with a purulent liquid as is seen in recent tubercular cavities. They often resembled moist plaster, or were quite dry and very friable, easily crumbling under the finger, or more dense, like a piece of chalk, and resisting the edge of the scalpel. It was not uncommon to find, in the midst of this substance, still harder portions, which were quite calcareous or sileceous. The color was not always of a uniform yellow, but often spotted with little black dots, or traversed by black lines. It was then evident that a portion of organized tissue entered into its composition. We once traced a number of bronchial branches into one of these calcareous masses, they were of a deep black, and after dividing several times terminated in their final twigs of the same color. In this as in analogous cases the matter adhered to the cyst, so that it was difficult to separate it.

Should we attempt, with a knowledge of the characters we have assigned to this chronic alteration of the lung, to trace out its origin, and indicate the kind of acute lesion to which it corresponds, we shall certainly conclude that it arose from an abscess, or from the softening of a mass of tubercles; most probably the latter, which is more frequent. Tubercles may exist in a limited portion of the lung, and their softening produce the morbid fluid contained in the cavity. We have remarked the frequent coindence of the two affections. Caverns, like the cysts or calcareous deposits, are most frequently situated at the summits of the lung—the slight difference in the structure of their walls may be explained by their various duration: in both cases, we generally find adhesions or other morbid lesions at the summit of the lungs, indicating previous inflammatory action.

The contained matter of both sometimes occur together, and they approximate each other so closely that it is often difficult to distinguish them: moreover, tubercular matter, when dessicated, resembles the calcareous substance above mentioned. In both cases we find remnants of organic tissues, indicating previous incomplete destruction of the pulmonary parenchyma, which occurred simultaneously with the suppuration, softening or transformation of the neighboring parts.

The puckering of the surface of the lung over these collections of matter, though not always found over tubercular caverns, may still serve to establish the identity of the morbid lesion in both cases. For what does it indicate but a retreat of the pulmonary tissue at this point? What does this shrinking determine? probably a diminished volume of the cyst, either from the absorption or condensation of the more fluid parts of the contained matter; and we have observed a similar puckering over tuberculous caverns which are diminishing. Laennec has noticed some cases of cicatrized ulcers in the lung.

From these considerations we are led to conclude that one of these alterations is a termination of the other, and that obliteration is also a curative effort of ulcers or tuberculous caverns. The cure, in this case, (and I here speak only of a purely local disorder,) would not be effected by cicatrization; but, *first;* because the softened tubercular matter that was retained, would not leave a fistulous cavity; *secondly;* because, when encysted and becoming a foreign body, this matter would be inert, and subject to absorption and to chemical decomposition.

If this be true, we understand why this lesion is more frequent in old subjects than young ones, the latter being generally cut off before any recuperative action could effect those organic alterations that require a long period for their development.

The smaller bronchiæ, like the little vessels near caverns or abscesses of the lung, are always obliterated by the action which induces softening or suppuration. The coat, or pseudo-membrane which lines them, passes over their orifice. I have sometimes observed a cylinder of plastic matter, passing like a plug a short distance within them. This is always observed in oblitered blood-vessels, even of large size; but is rarely seen in the greater air tubes which are here interrupted, on the contrary, they are ulcerated laterally; sometimes their walls are lined with an inorganic, soft whitish layer. I

have not observed cases enough of abscess to know whether the bronchiæ undergo the same changes, but we have already alluded to one in which the false membrane that lined the interior of the cavity, continued into the principal bronchia and induced its obliteration.

In phthisis, I have only observed two cases where the false membrane of the cavern was also continued five or six lines into the cavity of a large bronchia, though without obliterating it; further and more minute observation may prove this condition to be of more frequent occurrence.

I shall not now attempt to point out the period at which any obliteration has occurred, that may be observed near tubercular caverns: in abscess, I consider it probable that it results from the inflammatory action. Perhaps in some cases of tubercular softening, attended by evident inflammatory action, changes are produced in the same manner. At what stage soever the lesion may occur, we know that its effects ought to be the same, whether we refer the difficulty to the introduction of air, or the ulterior changes that may thence ensue in the substances contained in the morbid cavities.

### *Physiological Effects.*

To complete this subject, we must briefly advert to the consequences which these obliterations may produce in the lung itself. We have said that the neighboring bronchiæ were sometimes dilated, and that the surrounding pulmonary parenchyma was emphysematous. The organization of the lung is such that the various constituent parts cannot be supplied from one another: in the vascular system, on the contrary, an obstruction at any one point, can only produce a momentary suspension of the circulation; numerous anastomotic passages re-establish it, and carry the blood by other channels to the parts where it was impeded, and even to the lower portion of the obliterated vessel itself. Indeed the circulation seems temporarily increased, nor is it for some time restored to its natural condition. On the contrary, the entire absence of anastomoses in the bronchiæ, renders this transfer of air by collateral channels impracticable in all the parts to which the obliterated bronchia had been distributed; hence ensues a diminution in the extent of the respiratory surface, equivalent to the whole portion of the organ from which the air is excluded. Moreover, the inspiratory effort, or the force which introduces a given quantity of air into the whole lung, being undiminished, it produces the dilatation of the neighboring parts. Whence ensues a new source of diminished respiratory surface which keeps pace with the dilatation of the bronchia, and rarefaction of the pulmonary tissue.

If this obliteration were to exist at the same time in many bronchiæ, it would probably give rise to appreciable physiological effects; the respiratory murmur would no doubt be considerably modified in the parts near the alteration; though we are here little aided by auscultation. Among the symptoms observed in the patients, it would be difficult to discover which really belong to this lesion.

Having finished the first part of our work, we have now to speak of two other species of bronchial obliterations.

*Obliteration of the bronchiæ, in consequence of a substance formed and accumulated within them.*

Under this head we shall refer to the small bronchiæ and their terminations, having never observed large tubes occluded in this manner, though the small bronchial tubes are frequently filled and distended with matter of various character.

Sometimes the obliteration was confined to a small extent of the bronchia, and produced by a roundish mass; sometimes it continued along the tube filling all its divisions, even the terminal vesicles. It was either confined to a single branch or extended to all the branches of a lobe; whence resulted masses of altered pulmonary tissue: here the precise seat and nature of the lesion could not be determined by the external aspect of the lung, nor by a few cuts into its substance; it was necessary to dissect each separate branch, to discover that the general alteration resulted from the united obliteration of these several bronchiæ.

This obliterating matter resembled both tubercle and pseudo-membranes. In the first case, the consequent lesion offered characters of phthisis, producing granulations when isolated, and infiltration when diffused: in the second case there was a peculiar pneumonia or hepatization, which I shall here describe.

A man died at La Pitié in the last stages of an acute pneumonia, which had run its course very rapidly. At the autopsy, the left lung was found almost entirely hepatized, a portion of the summit only being sound. Cuts made in several directions presented a grained surface, which was red, interspersed with paler spots—at some points yellowish and black. In some places the color was more uniformly grey or like pus. The organ was covered with a recent, yellowish false membrane, that was not disturbed by opening the thorax. The density and specific gravity were the same as the liver: it was readily broken by pressure, and contained no air.

This was evidently a case of pneumonia in the second degree passing into the third or suppuration: as yet nothing was discovered to distinguish it from red or grey hepatization. But a more careful examination revealed cylinders of a tough substance jutting from the cut ends of the bronchiæ of the third and fourth order, and resembling the plugs of coagula found in blood-vessels. By making longitudinal incisions the bronchiæ were found to be filled with this substance forming a solid cylinder that followed the divisions even to the vesicles, and could be lifted out, so as to present the branched arrangement of a tree. These cylinders were generally very regular, except where they were garnished with small rounded lateral enlargements, (*renflemens*,) either isolated or clustered in festoons. Some of the cylinders presented these only on one side; to demonstrate them, it was only necessary to exert a slight traction upon the principal cord. Near the pleura these enlargements were more numerous, giving the divisions of the cords a knotted appearance, and immediately beneath this membrane their volume, form and color, precisely resembled the grains that appeared on a first glance at the lung, giving it, in the highest degree, the granulated aspect, which characterizes one of the forms of hepatization. The same thing was observed on the surface of the incisions: all the bronchiæ we axamined were affected—and they were filled inversely to their size; for where the plastic matter first appeared, it occupied less than a third of the bronchial calibre, whereas, when it approached their terminations it completely filled the cavity.

In external characters this substance resembled fibrine; it was a pale yellowish white, resisting, elastic and divisible into filaments. Under the microscope one of them seemed made up of globules like those of the blood. In the lateral and terminal swellings (*renflemens*) the colour was greyish, or stained with black. There were also some differences in those parts of the lung which were in the second or third stages of pneumonia: in the latter, the filaments were tarnished, moist, thinned and less resisting.

Nothing remarkable was observed in the texture of the bronchial walls, where these tubes were large enough to be examined, except that some of them seemed to tear more readily than in health. Towards their termination, many partook of the color of the contained matter, and some black lines were observed opposite to the swellings. The vessels were completely empty, and where they could be opened, white, without any evidence of inflammation. Finally, between the different terminal branches, there were red cellular spaces, that projected less than the greyish grains. In those parts hepatized to the third degree, the color of the grains and tissue was similar, but the former were less prominent and the latter was less granulated.

I have observed these alterations four times in subjects that had died of acute pneumonitis, and whose lungs presented only the characters of ordinary hepatization, upon the usual mode of examination. Further researches alone, can determine whether the silence of authors, and the paucity of our own observations, are attributable to the rarity of the lesion, or to an imperfect examination of the lungs. Obliteration of the bronchiæ by plastic matter, here constituted the main point of the alteration, and undoubtedly caused its severity. The last case ought also to be cited as proof of the true arrangement of the bronchiæ, since a cast was furnished of the ramifications.

A protracted and carefully digested consideration of the products that result from various morbid alterations, and of the changes which they may induce, has led us to believe that plastic lymph, the pseudo-membranes, the solid parts of the blood and fibrine itself, retained by any cause in the vessels, may, under peculiar conditions of the general economy, be transformed into various morbid products, without the necessity of any complicated organic apparatus being formed of the various parts. But, let us here ask, what would have become of the plastic matter in the bronchiæ of the cases just cited, had the patients been under the influence of a tubercular, schirrhous or other predisposition. Would not this case of pneumonitis, in a phthisical person, have produced some remarkable transformations in a lung, making it appear changed into a compact tuberculous mass? We know that such a condition is not uncommon in children, and we have often found it in the monkey of our menageries, an animal which is perhaps more subject than any other, in our climate, to pulmonary phthisis.

The following case of this kind is related to exhibit an alteration characterized by obliteration of the bronchiæ. The patient died under the care of M. Louis; he had presented symptoms of pulmonary phthisis. The left lung adhered throughout to the costal pleura, and intimate adhesions connected it with the pericardium. The pleura pulmonalis, over the whole extent of the superior lobe, formed a greyish white layer a line to a line and a half thick. A longitudinal section of this lobe showed a cavity in the upper half, the lower portion was transformed into a compact mass of a yellowish white color, firm, smooth on the incised surface, grained where torn, and yielding under the nail like crude, firm tubercular matter: the vesicular structure was destroyed, and, except the empty orifices of some large tubes, presenting no appreciable traces of blood-vessels or permeable bronchiæ. From the inner surface of the pleura, ran partitions of a greyish white color, as firm as the fibrous tissue, and which divided the mass into a number of separate compartments.

A minute dissection enabled us to make the following discoveries in the different anatomical elements of the organ the bronchiæ were free for a certain extent; their calibre appeared rather smaller than natural; the branches of the size of a large crow-quill, were partly filled with a yellowish white substance, which might be drawn out of the tubes by a slight force: this matter pervaded the smaller divisions of the bronchiæ, filling them more completely and even adhering in the small twigs, where it was confounded with them, so that it could only be demonstrated by pressing a portion of the lung between the fingers so as to make the matter spring out from the tubes. In the larger tubes this substance had little consistence, was soft, and of a paler color than the parenchyma; as we approached the bronchial extremities it became firmer, and its color more nearly resembled the neighboring parts. The walls of the larger air-tubes were not reddened, further on, they were transformed into tuberculous matter, and finally lost all of their natural characters except their canaliculate form. The same thing was observed in regard to the large bloodvessels, springing from the base of the lung, the isolated branches ran to separate points of the cavern, and were lost in the altered tissue at its surface: and many secondary branches were obliterated, and floated freely in the cavity. All the branches, when cut across were empty near their roots, further on, they contained coagula, their walls were covered with a layer of firm reddish tissue, that sensibly increased their thickness.

We know of no better title to designate this alteration than tuberculous croup of the lesser bronchiæ and vesicles. As in the preceding case, we found the bronchial tubes obliterated by being filled with solid matter, and their terminal divisions so changed as to resemble the matter they contained. We should also observe, that where it commenced in the bronchiæ of considerable size, this matter had not the tubercular appearance, and resembled the plastic matter of ordinary false-membranes.

I could report many cases where this kind of obliteration of the bronchiæ, from tubercular matter at circumscribed points of the organ, gives rise to granulations, and to one of the forms of anatomical lesion of phthisis. But, my observations on this subject having been made in consequence of communications from my friend, Dr. Carswell, I shall allow him to use the facts, with his own views of the primary seat of tuburcle in the lung.* Feeling confident that the subject will be treated with superior ability, I shall pass on to the third species of obliteration, that caused by compression.

### *Obliteration of the Bronchiæ by Compression.*

Many causes conspire to obstruct the air by compressing the passages, but I shall here confine myself to cases of complete obliteration. All tumours must produce this effect in the neighboring tubes: we have seen it effected by little calculous masses as large as peas, and here the occlusion continued to the end of the tubes.

We have not had time to assure ourselves that a number of the small bronchial tubes are not also obliterated in the disease described by Bayle as granular phthisis. This lesion would greatly diminish the field of respiration, when coincident with tubercles, the softening of which would obstruct the passage of air to other portions of the respiratory surface. I have not found obliteration from this cause in the large

* See Pathological Anatomy. Illustration of the elementary forms of disease; by R. Carswell, London, 1833; Nos. 1 to 6.

bronchial tubes and their primary branches, but have seen a tuburcular tumour developed around the first bronchia, whence arose all the branches of one lung. The subject was a monkey of the genus Macao, which had died of phthisis, after having been long kept in a menagerie. Some of the organs were highly tuberculous: such as the lungs, liver, spleen, mesentery and bronchial glands, and a mass was found in the pericardium. The left side of the thorax was decidedly shrunken, as it would have been after the absorption of a pleuretic effusion. The corresponding lung occupied much less space than usual, was quite void of air and looked as though it had been crowded against the vertebræ by an effusion: there was not, however, a trace of liquid, and the pleura was perfectly healthy and free from adhesions. Inflation of the lungs through the trachea dilated the right only. There was evidently a considerable obstruction at some portion of the left bronchus, which indeed could be penetrated but a short distance.

A rounded mass of tuberculous glands embraced this tube, which it had flattened and completely obstructed. When cut transversely, there appeared only a linear furrow, through which a slender scalpel handle might be introduced; beyond this the bronchus had its natural cavity, but, instead of being lubricated with the usual thin, frothy secretion, there was a reddish glairy mucous, without air-bubbles and resembling that often found in the neck of the uterus or in its cavity after death. This lung must have been quite impermeable to air during life. It may be asked why this obstacle, which prevented the entrance of air, did not also hinder its escape, and why that which was contained at the moment of complete obliteration, should not have remained incarcerated? We suppose that the air was insensibly absorbed, as is probably the case, to some extent, in health, and as is frequently observed in emphysema of the cellular tissue in all parts of the body. Another important fact observed in this case, is the shrinking of the thorax, in consequence of the obliteration of the bronchia which was destined to supply this lung with air. The ribs, though unattached by adhesions, were forced to follow the retreating lung, by the atmospheric pressure from without. I know of but one parallel case of shrinking in the human subject; perhaps a similar obliteration has never been seen. The fact just related, though belonging to comparative pathological anatomy should attract the attention of practitioners to this subject.

I shall now consider my task completed. The importance of the subject has perhaps been exceeded by the length of the memoir; but, I hope this will be obviated by the novelty and interest of the facts recorded.

